# Replication analysis of genetic association of the *NCAN-CILP2* region with plasma lipid levels and non-alcoholic fatty liver disease in Asian and Pacific ethnic groups

**DOI:** 10.1186/s12944-016-0181-z

**Published:** 2016-01-13

**Authors:** Supichaya Boonvisut, Kazuhiro Nakayama, Saho Makishima, Kazuhisa Watanabe, Hiroshi Miyashita, Munkhtulga Lkhagvasuren, Yasuo Kagawa, Sadahiko Iwamoto

**Affiliations:** Division of Human Genetics, Center for Molecular Medicine, Jichi Medical University, 3311-1 Yakushiji, Shimotsuke, Tochigi 329-0498 Japan; Jichi Medical University Health Care Center, Shimotsuke, Tochigi 329-0498 Japan; School of Medicine, Mongolian National University, Bayagol district, 11th khoroo, Ulaanbaatar-city, Mongolia; High Technology Research Center, Kagawa Nutrition University, Sakado, Saitama 350-0288 Japan

**Keywords:** TM6SF2, Asian and Oceanian populations, Triglyceride, Cholesterol, Fatty liver disease

## Abstract

**Background:**

The *Neurocan-cartilage intermediate layer protein 2 (NCAN-CILP2)* region forms a tight linkage disequilibrium (LD) block and is associated with plasma lipid levels and non-alcoholic fatty liver disease (NAFLD) in individuals of European descent but not in the Malay and Japanese ethnic groups. Recent genome-wide resequence studies identified a missense single-nucleotide polymorphism (SNP) (rs58542926) of the *transmembrane 6 superfamily member 2 (TM6SF2)* gene in the *NCAN-CILP2* region related to hepatic triglyceride content. This study aims to analyze the influences of SNPs in this region on NAFLD and plasma lipid levels in the Asian and Pacific ethnic groups and to reveal the reasons behind positive and negative genetic associations dependent on ethnicity.

**Methods:**

Samples and characteristic data were collected from 3,013 Japanese, 119 Palauan, 947 Mongolian, 212 Thai and 401 Chinese people. Hepatic sonography data was obtained from the Japanese individuals. Genotyping data of five SNPs, rs58542926, rs735273, rs1009136, rs1858999, and rs16996148, were used to verify the effect on serum lipid levels by multiple linear regression, and the association with NAFLD in the Japanese population was examined by logistic regression analysis.

**Results:**

rs58542926 showed significant association with the plasma triglyceride (TG) level in Japanese (*P* = 0.0009, effect size = 9.5 (±3.25) mg/dl/allele) and Thai (*P* = 0.0008, effect size = 31.6 (±11.7) mg/dl/allele) study subjects. In Mongolian individuals, there was a significant association of rs58542926 with total cholesterol level (*P* = 0.0003, 11.7 (±3.2) mg/dl/allele) but not with TG level. In multiple comparisons in Chinese individuals, rs58542926 was weakly (*P* = 0.022) associated with TG levels, although the threshold for statistical significance was not reached. In Palauan individuals, there was no significant association with the studied SNPs. rs58542926 also showed significant association with Japanese NAFLD. The minor allele (t) increased NAFLD risk (OR 1.682, 95 % CI 1.289–2.196, *p* value 0.00013).

**Conclusion:**

This study confirmed the genetic association of missense SNP of *TM6SF2*, rs58542926, with plasma lipid levels in multiple East Asian ethnic groups and with NAFLD in Japanese individuals.

## Background

Dyslipidemia, which refers to abnormally high or low lipid levels in circulating blood, is a major heritable risk factor of cardiovascular and cerebrovascular diseases. Many studies have investigated genetic factors of plasma lipid levels to explore the responsible metabolic pathways. Through genome-wide association studies (GWAS), abundant genetic variants associated with plasma lipid levels have been identified, among which the *Neurocan-cartilage intermediate layer protein 2 (NCAN-CILP2)* region has high statistical significance and a relatively large effect size per allele on low-density lipoprotein cholesterol (LDL) and triglyceride (TG) levels [1, 2]. The *NCAN-CILP2* region spans 300 kb on chromosome 19 and forms a tight linkage disequilibrium (LD) block. Eleven genes and one miRNA are encoded in this region. This locus showed consistent and deep association with serum lipid levels in the subsequent studies for individuals of European and Chinese descent [3–5]. However, the tag SNP in this region, rs16996148, was not correlated with plasma lipids in Malaysian [6] and Japanese populations [7, 8].

**Fig. 1:**
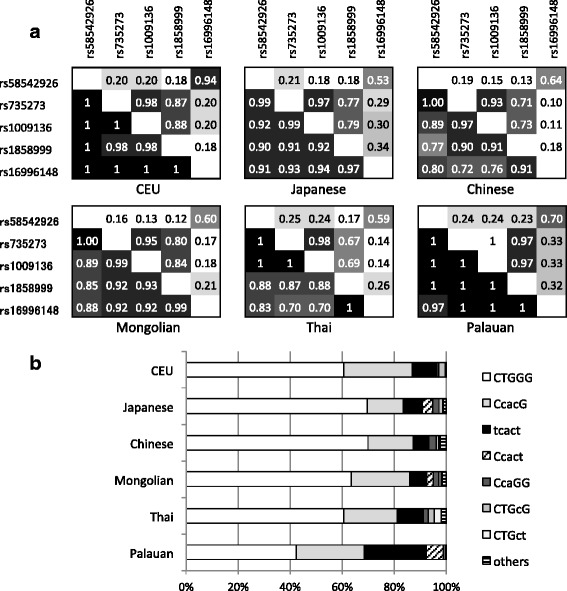
**a** Pairwise linkage disequilibrium analysis of the five studied SNPs in the *NCAN-CILP2* region in European (CEU), Japanese, Chinese, Mongolian, Thai and Palauan individuals. Numerals in the left-lower diagonal show |D’| values and in the right-upper diagonal half show r^2^ values. **b** Haplotype frequencies in the five studied populations and referential CEU population. The chart on the right shows the estimated haplotypes in the order of rs58542926, rs735273, rs1009136, rs1858999 and rs16996148. Capital letters indicate major alleles, and small letters indicate minor alleles

In addition to plasma lipid levels, the *NCAN-CILP2* region was identified as a non-alcoholic fatty liver disease (NALFD)–associated locus by GWAS in individuals of European descent [9] but not in Japanese individuals [10, 11]. The risk allele for NAFLD in this region decreased plasma lipid levels in individuals of European descent [9]. Recent genome-wide resequence studies identified a missense single-nucleotide polymorphism (SNP) (rs58542926) in *transmembrane 6 superfamily member 2 (TM6SF2)*, which is related to hepatic triglyceride content [12, 13]. Functional assessment of *Tm6sf2* in mouse and human hepatocytes revealed that it regulated plasma TG and cholesterol levels in mice and lipid accumulation in hepatocytes [12–14].

rs16996148 and rs58542926 are on opposite sides of the LD block. There was no significant difference in LD structure between European and Japanese populations in the HapMap database (http://hapmap.ncbi.nlm.nih.gov/). The present study aims to analyze the influences of SNPs in this region on NAFLD and plasma lipid levels in Asian and Pacific ethnic groups and to reveal the reasons behind positive and negative genetic correlations dependent on ethnicity.

## Results

### Genetic association of *NCAN-CILP2* region with East Asian plasma lipid levels

Genotyping success rates were >97 % for all of the SNPs. There were no deviations from Hardy-Weinberg equilibrium for any of the SNPs in all ethnic groups (*P* > 0.05). Details of all 5 SNPs and minor allele frequencies (MAFs) for each population are described in Table 1. rs58542926 showed significant associations with plasma lipid levels in multiple ethnic groups, which persisted after applying the Bonferroni correction for multiple testing (5 SNPs and 4 phenotypes). In Japanese individuals, rs58542926 was strongly associated with TG level. The minor allele (t) decreased the TG level by 9.5 (±3.25) mg/dl/allele. rs58542926, rs735273, rs1009136 and rs1858999 were weakly associated with TG level in Chinese individuals, but the associations did not reach the statistical significance threshold. In Mongolian individuals, a significant association of rs58542926 was observed with total cholesterol and LDL level, but not with TG level. The minor allele decreased total cholesterol by 11.7 (±3.2) mg/dl/allele and LDL cholesterol by 8.3 (±2.9) mg/dl/allele. However, the minor allele solely decreased the TG level in Thai individuals by 31.6 (±11.7) mg/dl/allele. In Palauan individuals, there were no significant associations with the studied SNPs. In combined analysis, the association of rs58542926 with total cholesterol and TG levels becomes further significant. These results support the previous data that the studied missense SNP in TM6SF2 is the functional SNP in the *NCAN-CILP2* region.

**Table 1:** Association study of SNPs in NCAN-CILP2 region with serum lipid levels in East Asian ethnic groups

dbSNP ID position Nearest gene Allele		Japanese (n = 3014)		Chinese (n = 401)		Mongol (n = 947)		Thai (n = 212)			Palau (n = 119)		Combined (n = 4693)	
rs58542926 chr19:19268740	MAF (*P*-HWE)	0.080 (0.858)		0.0682 (0.787)		0.078 (0.707)		0.114 (0.227)			0.237 (0.663)			
		*β*	*P* value	*β*	*P* value	*β*	*P* value		*β*	*P* value	*β*	*P* value	*β*	*P* value
TM6SF2	total cholesterol	−0.035	0.048	−0.089	0.0835	−0.116	0.0003*		−0.095	0.157	0.012	0.907	−0.051	0.0005*
E167K	LDL	−0.027	0.130	−0.041	0.4276	−0.091	0.005		−0.077	0.258	0.077	0.454	−0.030	0.039
C/t	HDL	0.005	0.747	0.0265	0.5837	−0.018	0.551		0.107	0.109	−0.128	0.195	−0.007	0.600
	Log TG	−0.054	0.0009*	−0.109	0.0224	−0.030	0.334		−0.222	0.0008*	−0.023	0.813	−0.056	3.15×10^-5*^
rs735273	MAf (*P*-HWE)	0.291 (0.719)		0.277 (0.613)		0.351 (0.788)			0.342 (0.151)		0.572 (0.966)			
chr19:19274602		*β*	*P* value	*β*	*P* value	*β*	*P* value		*β*	*P* value	*β*	*P* value	*β*	*P* value
TM6SF2	total cholesterol	0.008	0.674	−0.091	0.071	−0.034	0.293		−0.018	0.787	−0.049	0.639	−0.030	0.036
5’ flanking	LDL	−0.005	0.793	−0.019	0.700	0.001	0.986		0.024	0.724	−0.008	0.936	−0.019	0.181
T/c	HDL	0.037	0.019	−0.017	0.728	−0.038	0.211		0.024	0.721	−0.048	0.635	−0.005	0.716
	Log TG	−0.025	0.128	−0.098	0.038	−0.019	0.544		−0.147	0.028	−0.052	0.599	−0.031	0.019
rs1009136	MAF (*P*-HWE)	0.286 (0.757)		0.275 (0.706)		0.341 (0.911)			0.347 (0.128)		0.572 (0.966)			
chr19:19329619		*β*	*P* value	*β*	*P* value	*β*	*P* value		*β*	*P* value	*β*	*P* value	*β*	*P* value
MAU2	total cholesterol	0.008	0.663	−0.088	0.084	−0.042	0.186		−0.019	0.783	−0.049	0.693	−0.030	0.041
intronic	LDL	−0.005	0.793	−0.005	0.915	−0.001	0.977		0.021	0.755	−0.008	0.936	−0.016	0.275
G/a	HDL	0.038	0.018	−0.009	0.855	−0.041	0.178		0.036	0.590	−0.048	0.635	−0.005	0.719
	Log TG	−0.026	0.113	−0.094	0.047	−0.031	0.313		−0.148	0.027	−0.058	0.599	−0.035	0.010
rs1858999	MAF (*P*-HWE)	0.276 (0.844)		0.253 (0.349)		0.335 (0.422)			0.370 (0.168)		0.5812 (0.994)			
chr19:19386860		*β*	*P-*value	*β*	*P-*value	*β*	*P-*value		*β*	*P-*value	*β*	*P-*value	*β*	*P-*value
GATAD2A	total cholesterol	−0.003	0.853	−0.098	0.054	−0.047	0.139		−0.023	0.729	−0.053	0.610	−0.037	0.012
intronic	LDL	−0.019	0.289	−0.003	0.946	0.000	0.997		−0.008	0.909	−0.010	0.927	−0.022	0.121
G/c	HDL	0.046	0.004	0.008	0.866	−0.031	0.308		0.035	0.594	−0.067	0.501	0.001	0.997
	Log TG	−0.044	0.007	−0.110	0.021	−0.027	0.380		−0.112	0.096	−0.060	0.541	−0.042	0.002*
rs16996148	MAF (*P*-HWE)	0.119 (0.470)		0.069 (0.611)		0.099 (0.907)			0.132 (0.631)		0.308 (0.854)			
chr19:19547663		*β*	*P* value	*β*	*P* value	*β*	*P* value		*β*	*P* value	*β*	*P* value	*β*	*P* value
CILP2	total cholesterol	−0.034	0.057	−0.049	0.336	−0.092	0.004		−0.106	0.116	0.024	0.820	−0.032	0.026
3’ flanking	LDL	−0.032	0.069	0.005	0.923	−0.073	0.024		−0.094	0.166	0.123	0.238	−0.016	0.276
G/t	HDL	0.009	0.554	0.030	0.538	−0.049	0.106		0.091	0.171	−0.080	0.421	0.001	0.948
	Log TG	−0.038	0.020	−0.065	0.175	−0.007	0.817		−0.181	0.007	−0.058	0.552	−0.037	0.006

Beta coefficient (β) and *P* values of copy numbers of minor alleles for each lipid parameter in multiple linear regression models of the indicated ethnic groups are shown. Minor alleles of each SNP are indicated by lowercase letters. Statistical significance is shown by an asterisk (*) on the threshold of <0.0025. MAF (P HWE): minor allele frequency (*P* value of Hardy-Weinberg equilibrium.)

### Genetic association of the *NCAN-CILP2* region with Japanese NAFLD

Among the studied SNPs, rs58542926 showed the most significant association with Japanese NAFLD. The minor allele (t) increased NAFLD risk (OR 1.682, 95 % CI 1.289–2.196, *p* value 0.00013) (Table 2). The allele that reduced plasma lipid levels was the risk allele for NAFLD.

**Table 2:** Association study of SNPs in NCAN-CILP2 region with NAFLD in Japanese individuals

dbSNP ID (nearest gene)	OR	(95%CI)	*p* value
rs58542926 (TM6SF2)	1.682	(1.289–2.196)	0.001
rs735273 (TM6SF2)	1.290	(1.093–1.522)	0.003
rs1009136 (MAU2)	1.251	(1.060–1.478)	0.008
rs1858999 (GATAD2A)	1.199	(1.015–1.417)	0.033
rs16996148 (CILP2)	1.186	(0.943–1.493)	0.145

Odd ratio (OR) and P value of each minor allele in the logistic regression model are shown

### Haplotype frequency and linkage disequilibrium structures

To evaluate the LD structure of *NCAN-CILP2* in the Asian and Pacific populations, a pairwise linkage disequilibrium map was drawn for each ethnic group, and the maps were compared with |D’| and *r*^*2*^ values of the CEU population downloaded from the HapMap database (Fig. 1a). The middle three SNPs, rs735273, rs1009136, and rs1858999, showed relatively high *r*^*2*^ values in all of the ethnic groups. But, the most telomeric side SNP, rs58542926, showed weak LD with the middle LD block, even though the physical distance between rs58542926 and rs735273 was close, 5.8 kb, and they showed a high |D’| value, indicating rare recombinations between them. Conversely, the most centromeric side SNP, rs16996148, showed a moderate *r*^*2*^ score with rs58542926, despite the long distance between them, 289 kb. The *r*^*2*^ score between rs58542926 and rs16996148 in the CEU population was relatively high. The estimated haplotype frequency showed a higher prevalence of the haplotype encoding the NAFLD risk allele in the Palauan individuals as compared to other ethnic groups (Fig. 1b). Haplotypes carrying minor allele (t) of rs16996148 in the CEU population mostly (94.7 %) encoded as minor allele (t) of rs58542926, but in the Japanese, Mongolian, Thai and Palauan populations, substantial haplotypes (21.5–37.1 %) encoded major allele (C) of rs58542926 with (t) of rs16996148.

## Discussion

This study replicated the genetic association of a missense SNP in *TM6SF2* with plasma lipid levels in multiple ethnic groups, while the other four SNPs did not show significant associations. Lysine at residue 167 in *TM6SF2* reduced TG but did not reduce cholesterol in the plasma of Japanese and Thai individuals. In Mongolian individuals, rs58542926 reduced total cholesterol but not TG. Despite the higher minor allele frequency in Palauan individuals, rs58542926 was not associated with lipid levels. A small population size was considered as the main cause of the lack of association, but other ethnic-specific factors could have affected the results (e.g., relatively higher prevalence of type 2 diabetes (20.2 %) and obesity (87.8 %, body mass index (BMI) > 25)) in the Palauan population [15]. Population-dependent preference to associated lipid fraction was also observed in previous studies. Norwegians showed association only with total cholesterol, [12] but European Americans showed over 400-fold smaller p value with TG than LDL cholesterol [13]. The Hispanic group did not show any significant association with lipid levels [13]. These results suggest that the regulation of cholesterol and TG in plasma by *TM6SF2* is affected by ethnicity. The variable effect of rs58542926 on plasma lipid profiles may be feasibly explained by differences in dietary fat or neutraceutical intake [16] according to ethnic tradition.

This study also replicated the genetic association of *TM6SF2* with NAFLD in Japanese. The lysine allele of rs58542926 increased the risk diagnosed of a bright liver in ultrasonography. Although the exact biochemical function of *TM6SF2* protein has not yet been revealed, knocking down of *Tm6sf2* expression in mouse liver reduced cholesterol and TG levels in mouse plasma and increased hepatic lipid droplet content [12, 13]. In human hepatocyte cell lines, disturbed expression of *TM6SF2* reduced VLDL excretion and increased cytoplasmic lipid droplets [14]. Furthermore, amino acid substitution at residue 167 from glutamine to lysine diminished the protein level in the endoplasmic reticulum membrane of the transfected cells, probably via misfolded protein degradation [13]. Therefore, it was suggested that *TM6SF2* protein is involved in lipid transportation through the ER membrane. Our genetic results support the theory that the SNP induced a change of the physiological function of the *TM6SF2* gene.

rs16996148 was initially identified as an SNP associated with plasma cholesterol and TG in a population of European descent [2]. However, this finding was not replicated in Japanese individuals [7]. The LD score between rs16996148 and rs58542926 in the CEU population was more rigid than in East Asian populations, due to the low frequencies of Ccact and CTGct haplotypes. This distinct LD structure and haplotype frequencies in East Asian populations may have resulted in the lack of association. The haplotype frequency in our studied ethnic groups suggested that the functional SNP in *TM6SF2*, rs58542926, emerged in the haplotype encoding minor alleles at the remaining four SNPs. It was intriguing that the minor allele haplotype was highly prevalent in Palauan individuals as compared to other ethnic groups. Although rs58542926 is a risk allele for NAFLD, it has been reported to be beneficial for CAD [12, 17]. The *NCAN-CILP2* region was also suspected to be a type 2 diabetes–associated locus [18], although the pathogenic locus has not been identified. One explanation for these observations may be that the NAFLD risk allele was dramatically increased in the ancestors of Palauan individuals by natural selection, which would be related to energy metabolism. The NAFLD risk allele may be associated with a phenotype that stores excessive energy in the liver and might be advantageous under conditions of highly restricted food availability. Further population genetic studies are required to determine if the higher prevalence of the Lys167 allele in the Palauan population is a consequence of natural selection or simple genetic drift.

## Conclusion

In conclusion, our study replicated the genetic association of missense SNP of *TM6SF2*, rs58542926, with plasma lipid levels in East Asian multiple ethnic groups. While associated lipid, cholesterol, or TG levels were dependent on ethnicity, the effect sizes were relatively large. The SNP was also associated with Japanese NAFLD. These results support previous genetic studies and can be used to estimate the physiological function of *TM6SF2*, which could be a personal biomarker for medical management of dyslipidemia and NAFLD.

## Methods

### Study populations

The present study design was approved by the ethical committee of Jichi Medical University and Kagawa Nutrition University. All participants provided written informed consent. Samples were collected from 3,013 Japanese, 119 Palauan, 947 Mongolian, 212 Thai and 401 Chinese people. Population characteristics have been previously described [15, 19]. Participant age, sex, alcohol consumption (obtained by self-questionnaire), body mass index (BMI), and levels of plasma triglyceride (TG), total cholesterol, low-density lipoprotein cholesterol (LDL), and high-density lipoprotein cholesterol (HDL-c) (mg/dl) were collected. Hepatic sonography data were simultaneously obtained in Japanese samples for fatty liver evaluation, which was examined by an experienced clinical technologist and reviewed by clinicians.

### Genotyping and association analysis

The following five SNPs of the NCAN-CILP2 LD block were chosen considering the LD structure of the Japanese population and the results of recent studies [9–13]: rs58542926 (most adjacent gene; *TM6SF2*), rs735273 (*TM6SF2*), rs1009136 (*MAU2*), rs1858999 (*GATAD2A*), and rs16996148 (*CILP2*). All five SNPs were genotyped by the TaqMan Genotyping Assay Systems (Applied Biosystems, Foster City, CA, USA). Multiple linear regression analysis was employed to calculate the effect of each minor allele on four serum lipid concentration levels: TG, total cholesterol, LDL, and HDL. The analysis was calculated separately in each ethnic group. Age, sex, and BMI were included for adjustment as covariant factors. Triglyceride levels were transformed to logarithm to reduce the overestimated effect from skewed distribution, but untransformed values of triglycerides were also assessed for the purpose of showing an effect size of SNPs. To study the association of these five SNPs and NAFLD in the Japanese population, participants who had excessive alcohol consumption (>20 g/day) history were excluded. Then, genotyping results of 2,281 of the 3,013 Japanese DNA panels were studied. Logistic regression analysis was applied with adjustments for age, sex, BMI, and type 2 diabetes mellitus (DM2). All statistical analyses were performed with SPSS software. Haplotype frequencies for each population were calculated, and LD blocks were drawn by Haploview software [20].

## Abbreviations

BMI: body mass index
DM2: type 2 diabetes mellitus
GWAS: genome-wide association studies
HDL-c: high-density lipoprotein cholesterol
LD: linkage disequilibrium
LDL: low-density lipoprotein cholesterol
MAF: minor allele frequency
NAFLD: non-alcoholic fatty liver disease
NCAN-CILP2: Neurocan-cartilage intermediate layer protein 2
SNP: single-nucleotide polymorphism
TG: triglyceride
TM6SF2: transmembrane 6 superfamily member 2
